# Taxonomic note and description of new species of *Fissocantharis* Pic from China (Coleoptera, Cantharidae)

**DOI:** 10.3897/zookeys.443.8309

**Published:** 2014-09-29

**Authors:** Yuxia Yang, Junyan Su, Xingke Yang

**Affiliations:** 1 College of Life Sciences, Hebei University, Baoding 071002, Hebei Province, P. R. China; 2 Key Laboratory of Zoological Systematics and Evolution, Institute of Zoology, Chinese Academy of Sciences, Beijing 100101, China

**Keywords:** Taxonomy, *Fissocantharis*, synonym, homonym, new species, new name, China

## Abstract

Two new species of *Fissocantharis* Pic are described, *Fissocantharis bifoveatus* **sp. n.** (CHINA: Yunnan) and *Fissocantharis acuticollis* **sp. n.** (CHINA: Zhejiang, Fujian, Guangdong, Hunan). *Fissocantharis pieli* (Pic, 1937) is redescribed and *Fissocantharis kontumensis* Wittmer, 1989 is provided with a supplementary description. *Fissocantharis shanensis* (Wittmer, 1997) is synonymized with *Fissocantharis kontumensis*. For the above four species, illustrations of male genitalia are provided, for the latter three also photos of female genitalia and abdominal sternites VIII, and for the new species photos of male habitus and antennae are presented. Additionally, the specific name of *Fissocantharis wittmeri* (Y. Yang *et* X. Yang, 2009), preoccupied by *Fissocantharis wittmeri* (Kazantsev, 2007), is replaced by *Fissocantharis walteri* Y. Yang *et* X. Yang, nom. n. And *Fissocantharis wittmeri* (Kazantsev, 2007) is found to be a junior objective synonym of *Fissocantharis denominata* (Wittmer, 1997).

## Introduction

*Fissocantharis* Pic, 1921 is one of the largest genera of cantharid beetles, with about 200 species known worldwide, and the generic diagnosis was most recently redefined by Yang, Brancucci and Yang (2009). During our recent study, two remarkable new species of this genus from China were discovered. Here the new species are described under the names of *Fissocantharis bifoveatus* sp. n. and *Fissocantharis acuticollis* sp. n., which are related to *Fissocantharis pieli* (Pic, 1937) and *Fissocantharis kontumensis* Wittmer, 1989 respectively. A key to their similar species has been already provided by Yang, Okushima and Yang (2012), so only some differential diagnosis between each new species and its related species are summarized in the present study.

Furthermore, based on an examination of the types, *Fissocantharis shanensis* (Wittmer, 1997) (Type locality: Myanmar: Shan States), originally in *Micropodabrus* Pic, 1920, is considered to be a junior synonym of *Fissocantharis kontumensis* Wittmer, 1989 (Type locality: Vietnam: Kon Tum), which is recorded to China (Yunnan) for the first time.

Additionally, the specific name of *Fissocantharis wittmeri* (Y. Yang & X. Yang, 2009), a replacement name for *Rhagonycha coomani* Pic, 1935 (Type locality: Vietnam: Tonkin) and preoccupied by *Fissocantharis wittmeri* (Kazantsev, 2007), here is replaced by *Fissocantharis walteri* Y. Yang & X. Yang, nom. n. *Fissocantharis wittmeri* (Kazantsev, 2007), which was a replacement name for *Podabrus formosanus* Wittmer, 1954, is found to be an unnecessary replacement name and a junior objective synonym of *Fissocantharis denominata* (Wittmer, 1997).

In the present study, the characters of female genitalia and abdominal sternite VIII are emphasized in the description of *Fissocantharis* species for the first time.

## Material and method

The material is preserved in the following collections. Primary types were returned to the collections from which they were borrowed or were otherwise deposited in public museums.

**IZAS** Institute of Zoology, Chinese Academy of Sciences, Beijing, China;

**MHBU** Museum of Hebei University, Baoding, China;

**MNHN** Muséum national d’Histoire naturelle, Paris, France;

**NHMB** Naturhistorisches Museum Basel, Switzerland;

**SYSU** Sun Yat-Sen University, Guangzhou, China.

The genitalia of both sexes and abdominal sternites VIII of females were dissected and cleared in 10% KOH solution, and the female genitalia was dyed with hematoxylin. Habitus photos were taken by a Leica M205 A microscope, multiple layers were stacked using Combine ZM (Helicon Focus 5.3). Line drawings were made with the aid of camera lucida attached to a Leica MZ12.5 stereomicroscope, then edited in CorelDRAW 12 and Adobe Photoshop 8.0.1.

Complete label data are cited for type specimens, using square brackets “[ ]” for our remarks and comments, quotation marks to separate data from different labels.

Body length was measured from the anterior margin of the clypeus to the elytral apex and body width across the humeral part of elytra. Morphological terminology of female genitalia follows that of Brancucci (1980). The abbreviations in the figures are as follows, ag: accessory gland; co: coxite; di: diverticulum; tg9: abdominal tergite IX; sd: spermathecal duct; sp: spermatheca; ov: median oviduct; va: vagina.

## Taxonomy

### Fissocantharis pieli

Taxon classificationAnimaliaColeopteraCantharidae

(Pic, 1937)

Figs 3 , 6 , 11‒13

Lycocerus pieli Pic, 1937: 172.
Micropodabrus pieli : Wittmer 1997: 312, figs 178‒180.
Fissocantharis pieli : Yang et al., 2009: 49.

#### Type material examined.

Lectorype ♂ (MNHN): [p] “Mokan Shan \ 3.V.1930 \ coll. O. Piel”, [p] “LECTOTYPUS”, [h] “Lycocerus \ pieli n. sp.”, [h] “Micropodabrus \ pieli \ (Pic) \ det. W. Wittmer”. Paralectorype: 1♀ (MNHN): same data, 1.V.1930.

#### Additional material examined.

CHINA: **Zhejiang:** 2♂♂, 1♀ (IZAS): “Mokan Shan [Mogan Shan], 30.IV.1936, coll. O. Piel”; 1♀ (IZAS): same data, 3.V.1936; 1♀ (IZAS): Tianmu Shan, 6.V.1981, leg. P.Y. Yu; 2♂♂ (MHBU): Longquan, Fengyang Shan, 1250m, 31.III.2007, leg. J. Cao. **Fujian:** 1♂ (NHMB): “Fukien, Kuatun [Fujian, Guadun], 2300m, 27.40n.Br., 117.40ö.L., 5.IV.1938, J. Klapperich”; 1♂ (NHMB): same data, 30.III.1938; 1♂ (NHMB): “Fukien, Kuatun, 21.IV.1946, Tschung Sen.”; 1♂ (NHMB): “Fukien, Shaowu, Tachuland, 22.IV.1945”.

#### Redescription.

**Male.** Body black, clypeus and genae light brown, pronotum and elytra red, more or less darkened at median longitudinal groove of pronotum.

Head subquadrate, evenly narrowed behind eyes, dorsum slightly convex in center, with a distinct middle longitudinal line, each side with a small transverse impression behind antennal socket, head surface finely imbricate-punctate, matt, covered with sparse, fine, reddish brown decumbent pubescence; eyes moderately protruding, head width across eyes slightly wider than anterior margin of pronotum; terminal maxillary palpomeres nearly long-triangular, arcuate and sharp at apical one-third length of inner margin; antennae extending to apical one-third length of elytra, antennomeres II nearly as long as wide at apices, III‒VIII distinctly and IX‒X slightly widened apically, slightly flattened on dorsal sides, III about twice as long as wide at apices, IV slightly longer than III, the whole length of III‒V and basal parts of VI with narrow longitudinal ridges along outer margins, VI‒VIII each with a deep and nearly oblong fovea on dorsal side, the foveae slightly widened apically and smooth on inner surface, with all margins delimitated and well-developed on VI‒VII, but apical margins reduced on VIII, XI slightly longer than X, nearly parallel-sided and pointed at apex.

Pronotum subquadrate, slightly longer than wide, widest near posterior margin, anterior margin arcuate, anterior angles widely rounded, lateral margins slightly sinuate, moderately diverging posteriorly, posterior angles obtusely rectangular, posterior margin nearly straight and narrowly bordered, disc moderately convex on posterolateral parts, with a distinct median longitudinal groove, surface pubescent and punctate like that of head.

Elytra about 4.5 times longer than pronotum, 3.5 times longer than humeral width, which about one-third wider than posterior margin of pronotum, outer margins nearly parallel, disc surface rugulose-lacunose, densely and coarsely punctate, matt, covered with dense, short and decumbent reddish brown pubescence, combined with much sparser, longer, semierected pubescence, elytral venation well developed.

All claws bifid, the lower claws nearly as long as upper ones on proclaws, distinctly shorter than on meso- and metaclaws.

Abdominal sternite IX nearly triangular. Aedeagus (Figs 11‒13): ventral process of each paramere abruptly narrowed apically and rounded at apex; conjoint dorsal fig of parameres distinctly shorter than ventral processes, slightly emarginated in middle of apical margin and lateroapical angles; middle node of basal pieces moderately diverging apically.

**Female.** Similar to males, but antennae shorter and wider, extending to elytral midlength, antennomeres III‒X nearly triangular, each about 1.5 times as long as wide at apex, III‒V slightly and VI‒VIII distinctly concaved on dorsal sides, without delimitated margins and not smooth on inner surface. Pronotum nearly as wide as long, slightly convex on posterolateral parts. Elytra with outer margins slightly diverging posteriorly. Legs with all lower claws distinctly shorter than upper ones. Abdominal sternite VIII (Fig. 3) narrowly rounded at apex, hardly emarginated in middle of posterior margin. Internal reproductive organ of genitalia (Fig. 6): vagina stout and abruptly extended apically as a thin and long duct; diverticulum and spermathecal duct arising from the end the long duct of vagina; diverticulum moderately long, thin and spiral; spermathecal duct slightly shorter than diverticulum; spermatheca slightly thicker and longer than diverticulum, provided with moderately long and thin accessory gland, distinctly longer than spermatheca.

Body length: 10.0‒12.0 mm; width: 2.0‒2.5 mm.

#### Distribution.

China (Zhejiang, Fujian).

#### Remarks.

The characteristic antennae and aedeagus were illustrated by Wittmer (1997), but other morphological characters are poorly known except the simple description in the original publication (Pic, 1937). Under this consideration, we redescribe this species here and provide illustrations of its main diagnostic characters.

### Fissocantharis bifoveatus

Taxon classificationAnimaliaColeopteraCantharidae

Y. Yang & X. Yang sp. n.

http://zoobank.org/BE256688-BCA0-4C42-A1EE-183EF86563D3

Figs 1 , 9 , 14‒16

#### Type material.

Holotype ♂ (IZAS): “**CHINA, Yunnan** Provin., Gongshan, Dulongjiang, Miliwang, above Bapo, 27.72383°N, 98.36117°E, 1956m, 31.X.2004, night, Liang Hongbin collector, California Academy & IOZ, Chinese Acad. Sci.”. Paratypes: 1♂ (IZAS): same data to holotype; 1♂ (IZAS): “CHINA, Yunnan Provin., Gongshan, Dulongjiang, Kongdang, roadside, 27.87696°N, 98.33587°E, 1525m, 25.X.2004, day, Liang Hongbin collector, California Academy & IOZ, Chinese Acad. Sci.”; 1♂ (IZAS): “CHINA, Yunnan Provin., Gongshan, Bingzhongluo, Chaohong Bridge, beach, 28.06671°N, 98.58360°E, 1540m, 11.XI.2004, day, Liang Hongbin collector, California Academy & IOZ, Chinese Acad. Sci.”.

#### Description.

**Figures 1–5.**
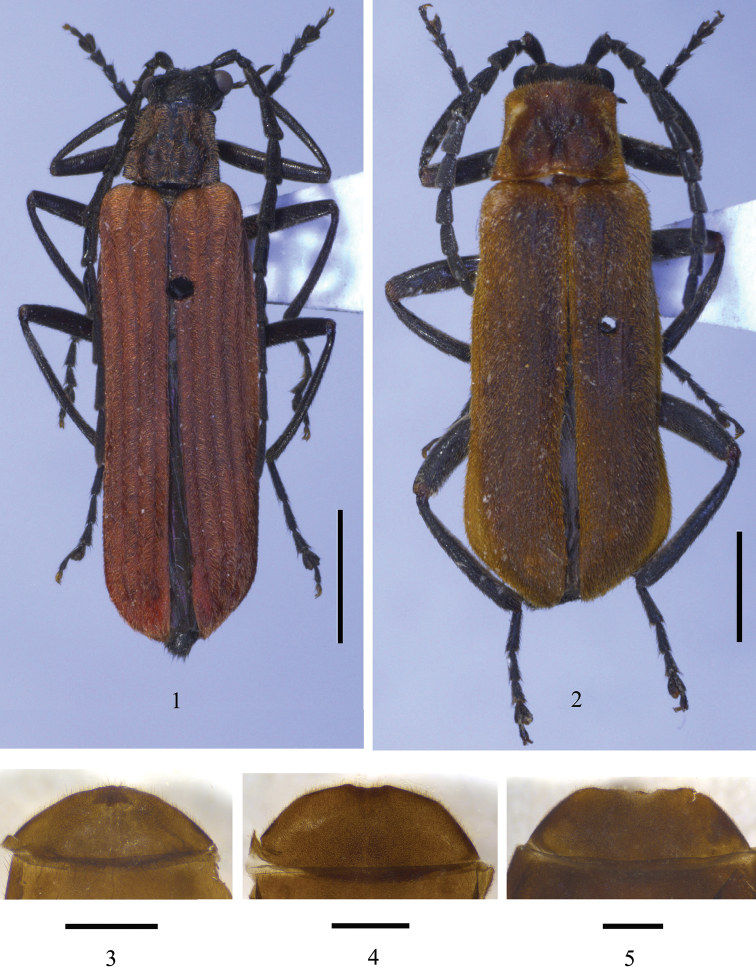
**1–2** Male habitus, dorsal view **3–5** abdominal sternite VIII of female, ventral view: **1** *Fissocantharis bifoveatus* sp. n. **2, 5** *Fissocantharis acuticollis* sp. n. **3** *Fissocantharis pieli* (Pic, 1937) **4** *Fissocantharis kontumensis* Wittmer, 1989. Scale bars: **1–2**=2.0 mm; **3–5**=1.0 mm.

**Male** (Fig. 1). Body black, mandibles dark brown, elytra red.

Head subquadrate, evenly narrowed behind eyes, dorsum slightly convex in center, with a distinct middle longitudinal line, each side with a small transverse impression behind antennal socket, head surface finely imbricate-punctate, matt, covered with sparse, fine, reddish brown decumbent pubescence; eyes moderately protruding, head width across eyes slightly wider than anterior margin of pronotum; terminal maxillary palpomeres nearly long-triangular, arcuate at apical one-third length of inner margin; antennae (Fig. 9) extending to apical one-third length of elytra, antennomeres II nearly as long as wide at apices, III‒VIII distinctly widened apically, slightly flattened on dorsal sides, III‒VII with outer apical angles distinctly protruding, III about twice as long as wide at apices, IV slightly longer than III, IV‒VI (Fig. 9a) each with a small, round, shallow impression at basal one-third of dorsal side, the whole length of IV‒VI and basal parts of VII with narrow longitudinal ridges along inner margins, VII‒VIII (Fig. 9b) each with a deep oblong fovea on dorsal side, the foveae smooth on inner surfaces, with all margins delimitated and well-developed, IX‒XI nearly parallel-sided, XI slightly longer than X and pointed at apex.

Pronotum subquadrate, slightly longer than wide, widest near posterior margin, anterior margin arcuate, anterior angle widely rounded, lateral margins slightly sinuate, moderately diverging posteriorly, posterior angles nearly rectangular, posterior margin nearly straight and narrowly bordered, disc moderately convex on posterolateral parts, with a distinct median longitudinal groove, surface pubescent and punctate like that of head.

Elytra about 4.5 times longer than pronotum, 3.5 times longer than humeral width, which about one-third wider than posterior margin of pronotum, outer margins nearly parallel, disc surface rugulose-lacunose, densely and coarsely punctate, matt, covered with dense, short and decumbent reddish brown pubescence, combined with much sparser, longer, semierected pubescence, elytral venation well developed, moderately costate.

All claws bifid, the lower claws nearly as long as upper ones at pro- and mesoclaws, slightly shorter than at metaclaws.

Abdominal sternite IX nearly triangular at apex. Aedeagus (Figs 14‒16): ventral process of each paramere slightly narrowed apically and rounded at apex, with inner margins curling up outwards; conjoint dorsal fig of parameres distinctly shorter than ventral processes, largely and triangularly emarginated in middle of apical margin, lateroapical angles obtusely triangular; middle node of basal pieces strongly diverging apically.

**Female.** Unknown.

Body length (males): 8.0‒9.0 mm; width: 1.8‒2.0 mm.

#### Diagnosis.

This species is similar to *Fissocantharis pieli* (Pic), but differs from the latter by the characteristic antennae of the male with antennomeres IV‒VI each with a small, round, shallow impression at basal one-third of dorsal side, VII‒VIII each with a deep oblong fovea on dorsal side; pronotum black; aedeagus: ventral process of each paramere slightly narrowed apically, with inner margins curling up outwards; conjoint dorsal fig of parameres largely and triangularly emarginated in middle of apical margin.

#### Distribution.

China (Yunnan).

#### Etymology.

The specific name is derived from the Latin *bi* (two) and *fovea* (pit), referring to its antennomeres VII‒VIII each with a deep fovea on dorsal sides in males.

**Figures 6–8.**
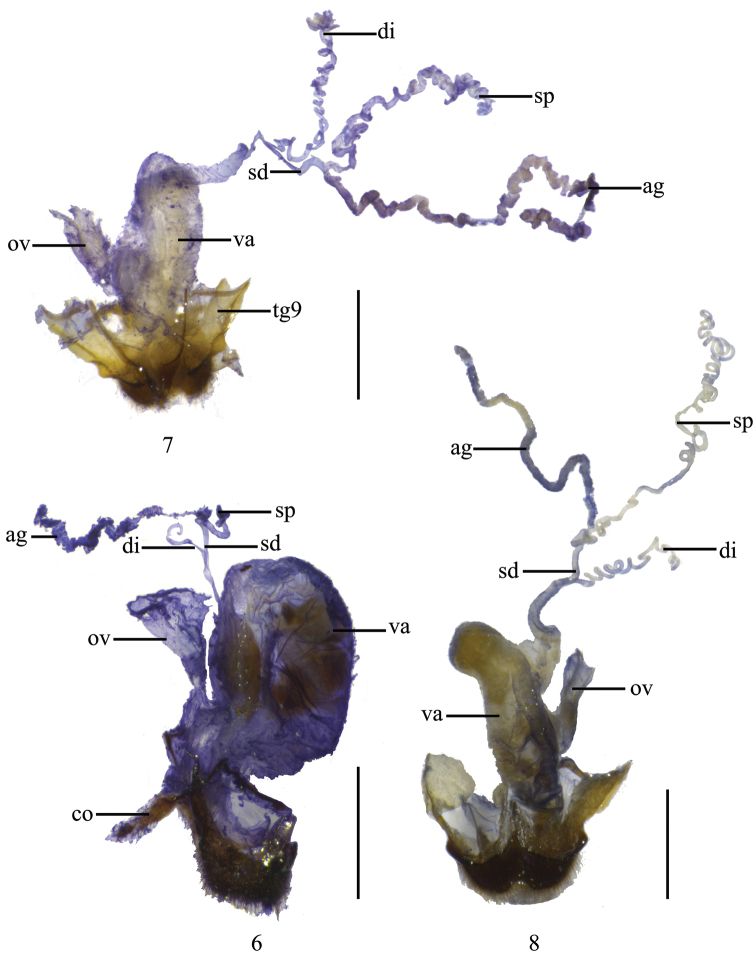
Female genitalia, lateral view: **6** *Fissocantharis pieli* (Pic, 1937) **7** *Fissocantharis kontumensis* Wittmer, 1989 **8** *Fissocantharis acuticollis* sp. n. Scale bars: 1.0 mm.

### Fissocantharis kontumensis

Taxon classificationAnimaliaColeopteraCantharidae

Wittmer, 1989

Figs 4 , 7 , 17‒19

Fissocantharis kontumensis Wittmer, 1989: 215, figs 12, 13.
Micropodabrus shanensis Wittmer, 1997: 313, figs 181, 182. syn. n.
Fissocantharis shanensis : Yang et al., 2009: 49.

#### Type material examined.

*Fissocantharis kontumensis*: Holotype ♂ (NHMB): [p] “VIETNAM: Buon-loi \ 40 km N of Ankhe \ Prov. Gia. Lai Kontum \ 12.–14.6.1985”, [p] “HOLOTYPUS”, [h] “F. \ kontumensis \ Wittm. \ det. W. Wittmer”, [p] “Naturhist. \ Museum Basel \ coll. W. Wittmer”, [p] “CANTHARIDAE \ CANTH00001272”.

*Micropodabrus shanensis*: Holotype ♂ (NHMB): [p] “S. SHAN States \ Burma 1500m. \ Taunggyi 1.VIII- \ 22.IX.(19)34 Malaise”, [p] “HOLOTYPUS”, [h] “Micropodabrus \ shanensis \ Wittm. \ det. W. Wittmer”, [p] “Naturhist. \ Museum Basel \ coll. W. Wittmer”, [p] “CANTHARIDAE \ CANTH00000259”. (The antennomeres IX-XI, right prometarsomeres IV-V, left metatarsus of the holotype were missing.)

#### Additional material examined.

**CHINA: Yunnan:** 1♂ (IZAS): Xishuangbanna, Mengzhe, 1200m, 29.VIII.1958, leg. F.J. Pu; 1♂ (IZAS): Xishuangbanna, Mengla, 620‒650m, 10.VI.1959, leg. F.J. Pu; 1♂, 2♀♀ (IZAS): Xishuangbanna, Meng’a, 1050‒1080m, 4.VIII.1958, leg. S.Y. Wang; 1♀ (IZAS): Lancang, 1000m, 26.VII.1957, leg. L.C. Zang; 1♂ (IZAS): same locality, 25.VII.1957, leg. S.Y. Wang; 1♂ (IZAS): Jinping, Changpotou, 1300m, 25.V.1956, leg. K.R. Huang; 1♀ (MHBU): Longling, Lameng, 3.VIII.2005, leg. B.Y. Mao & J.S. Xu.

#### Supplementary description.

**Male.** Aedeagus (Figs 17‒19): ventral process of each paramere slightly widened and rounded at apex; conjoint dorsal fig of parameres distinctly shorter than ventral processes, largely roundly emarginated in middle of apical margin, lateroapical angles slightly acute; middle node of basal pieces moderately diverging apically.

**Female.** Abdominal sternite VIII (Fig. 4) slightly truncate at apex, bisinuately emarginated in middle of posterior margin. Internal reproductive organ of genitalia (Fig. 7): vagina abruptly extended apically as a long and thick duct; diverticulum and spermathecal duct arising from the end the long duct of vagina; diverticulum long, thin and spiral; spermathecal duct distinctly shorter and slightly thicker than diverticulum; spermatheca nearly as long as and slightly thicker than diverticulum, provided with moderately long and thin accessory gland, slightly longer than spermatheca.

Body length: 8.0‒10.0 mm; width: 2.0‒2.2 mm.

#### Distribution.

China (new record: Yunnan); Vietnam; Myanmar.

#### Remarks.

*Fissocantharis shanensis* (Wittmer, 1997) was described on a single male holotype. Although some differential characters from *Fissocantharis kontumensis* Wittmer, 1989 were suggested by Wittmer (1997), these differences of quantitative change in the antenna and conjoint dorsal fig of parameres of aedeagus turned out to be intraspecific variability, based on our examination of both types and a large series of additional material. Therefore, we synonymize *Fissocantharis shanensis* (Wittmer, 1997) with *Fissocantharis kontumensis* Wittmer, 1989 here.

**Figures 9–10.**
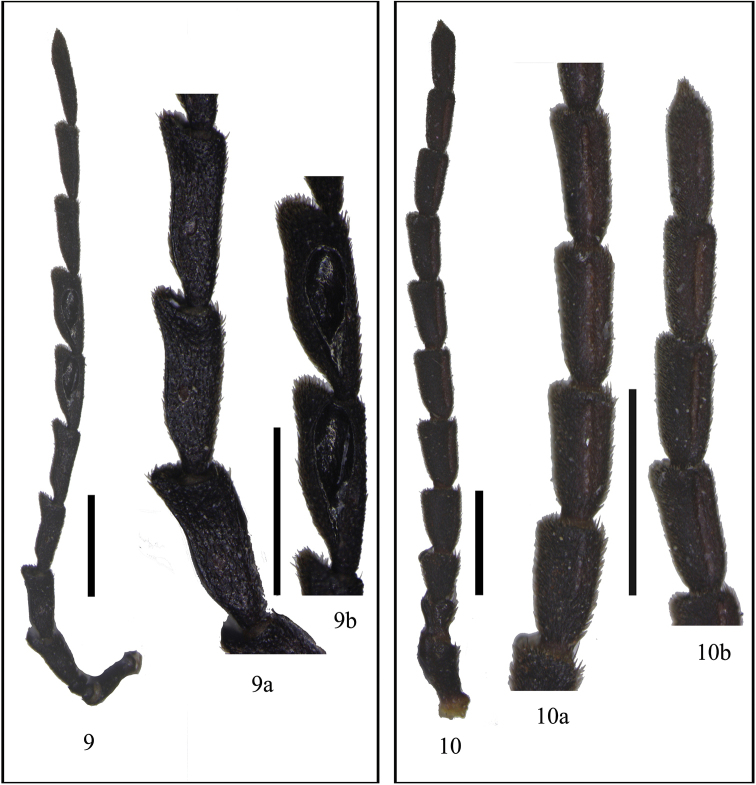
Male antennae, dorsal view: **9** *Fissocantharis bifoveatus* sp. n. (**9a** antennomeres IV– VI, dorsal view **9b** antennomeres VII– VIII, dorsal view) **10** *Fissocantharis acuticollis* sp. n. (**10a** antennomeres IV– VII, outer view **10b** antennomeres VIII– XI, outer view). Scale bars: 1.0 mm.

**Figures 11–16.**
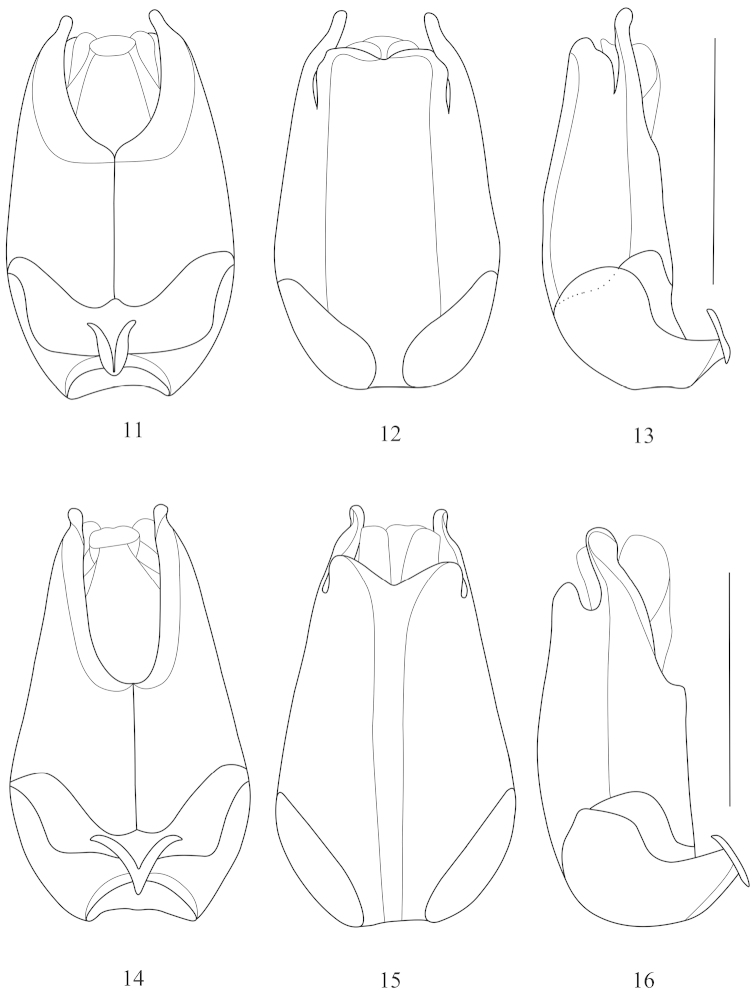
Aedeagus (**11, 14** ventral view **12, 15** dorsal view **13, 16** lateral view): **11–13** *Fissocantharis pieli* (Pic, 1937) **14–16** *Fissocantharis bifoveatus* sp. n. Scale bars: 1.0 mm.

### Fissocantharis acuticollis

Taxon classificationAnimaliaColeopteraCantharidae

Y. Yang & X. Yang sp. n.

http://zoobank.org/5BD2F700-8AAB-4375-8C80-248AA1BC0224

Figs 2 , 5 , 10 , 20‒22

#### Type material.

Holotype ♂ (MHBU): **CHINA: Zhejiang:** 1Taishun, Wuyanling, 28.VII‒3.VIII.2005, leg. Y.B. Ba. Paratypes: 1♀ (MHBU): same data as the holotype; 1♀ (MHBU): same data, 2.VIII.2005; 1♀ (MHBU): same data, 31.VII.2005; 1♀ (IZAS): Qingyuan, Baishanzu, 800m, 14.VIII.1993, leg. H. Wu. **Guangdong:** 1♀ (SYSU): Xinfeng, 10.VII.1991, leg. R. Zeng; 1♂ (SYSU): same locality, 8.VII.1991, leg. Q.Z. Ye; 1♀ (SYSU): same locality, 9.VII.1991, leg. R. Chen; 1♂ (SYSU): same locality, 9.VII.1991, leg. Z.Y. Weng; 1♂ (SYSU): same data, 8.VII.1991; 1♂ (SYSU): Fengkai, Heishiding, 18.‒22.VII.2007, leg. L. Shi; 1♂ (SYSU): same locality, 4.VII.1987, leg. S.X. Zhong; 1♀ (SYSU): same locality, 12.VII.1999, leg. J.N. Yang; 1♀ (IZAS): Ruyuan, Nanling Nature Reserve, Ruyang Reserve Station, 1030‒1420m, 20.VII.2008, leg. G.Y. Yang. **Hunan:** 1♂ (IZAS): Shennong Valley, Shennong Waterfall, 600‒900m, 7.VII.2008, leg. X.Y. Zhu & Z. Yang; 1♀ (IZAS): Yanling, Shidu, Taoyuandong, 868m, 26.478°N, 114.04°E, 7.VII.2008, T.Y. Jiao; 1♂ (IZAS): Yizhang, 14.‒15.VII.2008, leg. R.R. Wang & L. Ding (above all transliterated from Chinese labels). **Fujian:** 1♂ (NHMB): “Guatun, Fukien, China, 8.V.1946 (Tschung Sen.)”; 1♀ (NHMB): “Yen-ping, China, 2.VII.1917, Ac. 5148”.

#### Description.

**Male** (Fig. 2). Body black, clypeus and mouthparts except maxillary and labial palpi dark brown, pronotum and elytra yellowish brown.

Head subquadrate, evenly narrowed behind eyes, dorsum nearly flat, with a indistinct middle longitudinal line, each side with a small transverse impression behind antennal socket, head surface densely and finely punctate, semilustrous, covered with sparse, fine, yellowish brown decumbent pubescence; eyes moderately protruding, head width across eyes nearly as wide as anterior margin of pronotum; terminal maxillary palpomeres nearly long-triangular, almost obliquely truncate at apical one-third length of inner margin; antennae (Fig. 10) extending to basal one-third length of elytra, antennomeres II nearly as long as wide at apices, III‒XI distinctly thickened, III‒X widened apically, III nearly as long as wide at apices, IV slightly longer than III, VI longest, apical parts of IV, the whole length of V‒X and basal parts of XI with longitudinal deep grooves along outer margins (Figs 10a, b), XI nearly parallel-sided and pointed at apex.

Pronotum nearly trapeziform, distinctly wider than long, widest at posterior margin, anterior margin slightly arcuate, anterior angles obtusely rectangular, lateral margins nearly straight, strongly diverging posteriorly, posterior angles triangular and sharp, posterior margin nearly straight and narrowly bordered, slightly emarginated in middle, disc strongly convex on posterolateral parts, with a distinct median longitudinal groove, surface finely imbricate-punctate, matt, covered with dense, fine, yellowish brown decumbent pubescence.

Elytra about 4.0 times longer than pronotum, 2.5 times longer than humeral width, which about one-third wider than posterior margin of pronotum, outer margins slightly diverging posteriorly, disc surface rugulose-lacunose, densely and coarsely punctate, matt, covered with dense, short and decumbent reddish brown pubescence, combined with much sparser, longer, semierected pubescence, elytral venation moderately developed.

All claws bifid, the lower claws slightly shorter than upper ones on all claws.

Abdominal sternite IX nearly triangular at apex. Aedeagus (Figs 20‒22): ventral process of each paramere bent inwards and rounded at apex, with a triangular protuberance at inner margin; conjoint dorsal fig of parameres nearly as long as ventral processes, distinctly roundly emarginated in middle of apical margin, lateroapical angles rounded; middle node of basal pieces strongly diverging apically.

**Female.** Similar to males, but antennae slightly thickened, without longitudinal grooves on antnnomeres VI‒ XI. Head width across eyes distinctly narrower than anterior margin of pronotum. All claws with lower claws distinctly shorter than upper ones. Abdominal sternite VIII (Fig. 5) largely truncate at apex, bisinuately emarginated on each side of posterior margin. Internal reproductive organ of genitalia (Figs 20‒ 22): vagina tapered and extended apically as a long duct; diverticulum and spermathecal duct arising from the end the long duct of vagina; diverticulum moderately long, thin and spiral; spermathecal duct distinctly shorter and slightly thicker than diverticulum; spermatheca much longer than diverticulum, provided with moderately long and thin accessory gland, distinctly shorter than spermatheca.

Body length: 9.0‒11.0 mm; width: 2.3‒3.0 mm.

#### Diagnosis.

This species is similar to *Fissocantharis kontumensis* Wittmer, but differs from the latter by the characteristic antennae of the male with apical parts of antennomeres IV and the whole length of V with longitudinal grooves along outer margins; pronotum distinctly wider than long, with triangular and sharp posterior angles, disc strongly convex on posterolateral parts; aedeagus: ventral process of each paramere with inner margins distinctly protuberant in middle, conjoint dorsal fig of parameres nearly as long as ventral processes, roundly emarginated in middle of apical margin, with rounded lateroapical angles.

#### Distribution.

China (Zhejiang, Fujian, Guangdong, Hunan).

#### Etymology.

The specific name is derived from the Latin *acutus* (acute) and *collum* (neck), referring to its pronotum with triangular and sharp posterior angles.

#### Remarks.

Some specimens are variable, with head almost yellowish brown, pronotum and elytra more or less darkened, elytral venation hardly visible.

**Figures 17–22.**
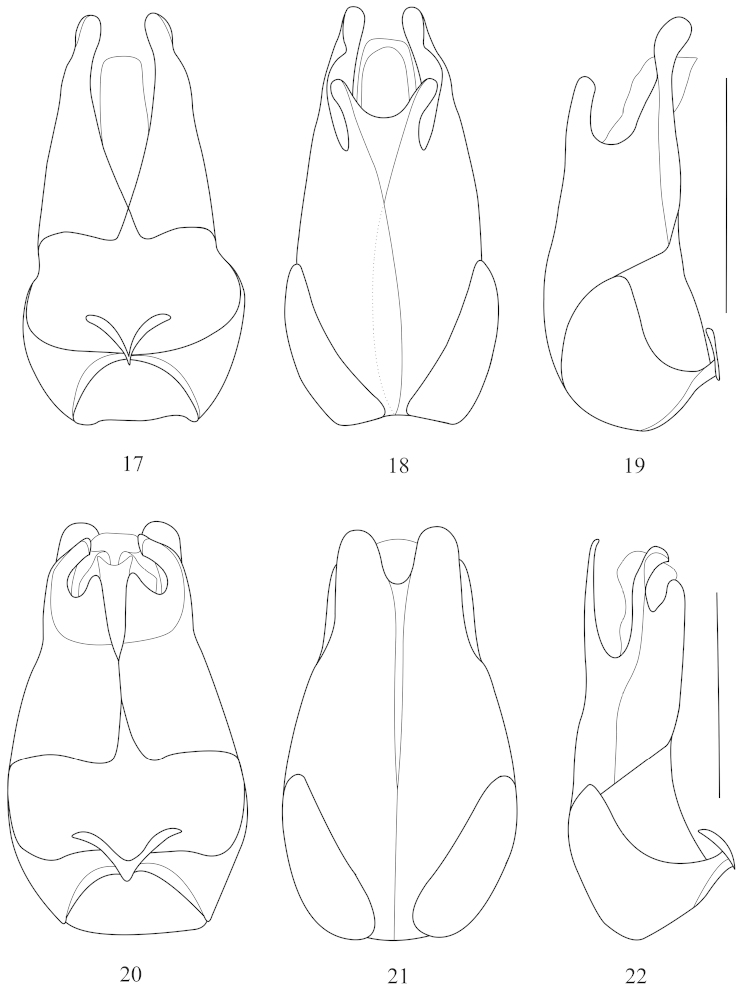
Aedeagus (**17, 20** ventral view **18, 21** dorsal view **19, 22** lateral view): **17–19** *Fissocantharis kontumensis* Wittmer, 1989 **20–22** *Fissocantharis acuticollis* sp. n. Scale bars: 1.0 mm.

### Fissocantharis walteri

Taxon classificationAnimaliaColeopteraCantharidae

Y. Yang & X. Yang nom. n.

Rhagonycha coomani Pic, 1935: 12.
Kandyosilis coomani : Wittmer 1989: 226.
Micropodabrus coomani : Wittmer 1997: 312 [secondary hononym, preoccupied by *Micropdabrus coomani* (Pic, 1926: 35), originally in *Lycocerus*].
Micropodabrus wittmeri Y. Yang & X. Yang, 2009: 67 (replacement name for *Micropodabrus coomani* (Pic, 1935), nec Pic, 1926) [primary homonym, preoccupied by *Micropodabrus wittmeri* Kazantsev, 2007]. syn. n.
Fissocantharis wittmeri Y. Yang & X. Yang, 2009: Yang et al. 2009: 49.

#### Distribution.

Vietnam (Tonkin).

#### Etymology.

The species is named after the first name of late Dr. Walter Wittmer.

#### Remarks.

This species was originally described in *Rhagonycha* Eschescholtz, 1830, and initially transferred to *Kandyosilis* Pic, 1929 (Wittmer, 1989), then later to *Micropodabrus* (Wittmer, 1997), where it became a junior secondary homonym of *Micropdabrus coomani* (Pic, 1926), which was originally in *Lycocerus* Gorham, 1889, so its specific name was replaced by *Micropodabrus wittmeri* Y. Yang & X. Yang, 2009. However, the latter had been preoccupied by *Micropodabrus wittmeri* Kazantsev, 2007, so *Micropodabrus wittmeri* Y. Yang & X. Yang, 2009 is permanently invalid as a junior homonym (ICZN 4^th^, Article 57.2) and must be replaced by a new substitute name (ICZN 4^th^, Article 60.1). Now this species is placed in *Fissocantharis* Pic, 1921, so a replacement name is proposed here as *Fissocantharis walteri* Y. Yang & X. Yang, nom. n.

### Fissocantharis denominata

Taxon classificationAnimaliaColeopteraCantharidae

(Wittmer, 1997)

Podabrus formosanus Wittmer, 1954: 274.
Micropodabrus taiwanus Wittmer, 1982: 130 [replacement name for *Podabrus formosanus* Wittmer, 1954].
Micropodabrus denominatus Wittmer, 1997: 310 [replacement name for *Micropodabrus taiwanus* Wittmer, 1982, nec Wittmer, 1979].
Micropodabrus wittmeri Kazantsev, 2007: 54 [replacement name for *Micropodabrus taiwanus* Wittmer, 1982, nec Wittmer, 1979]. syn. n.
Fissocantharis denominata : Yang et al. 2009: 49.
Fissocantharis wittmeri Kazantsev: Yang et al. 2009: 49.

#### Distribution.

China (Taiwan).

#### Remarks.

Kazantsev (2007) proposed *Micropodabrus wittmeri* as a replacement name for *Micropodabrus formosanus* (Wittmer, 1954), which was originally described in *Podabrus* Westwood, 1838. However, *Micropodabrus formosanus* had been already replaced by a replacement name as *Micropodabrus denominatus* Wittmer, 1997, so *Micropodabrus wittmeri* Kazantsev, 2007 is a junior objective synonym of *Micropodabrus denominatus*, which is the valid name as the oldest available name applied to this species (ICZN 4^th^, Article 57.2).

## Supplementary Material

XML Treatment for Fissocantharis pieli

XML Treatment for Fissocantharis bifoveatus

XML Treatment for Fissocantharis kontumensis

XML Treatment for Fissocantharis acuticollis

XML Treatment for Fissocantharis walteri

XML Treatment for Fissocantharis denominata
